# A case of a transplanted kidney with an orthotopic kidney stone

**DOI:** 10.1093/jscr/rjae445

**Published:** 2024-08-29

**Authors:** Zhaofang Jin, Jianjun Lai, Jianjun Zhang

**Affiliations:** Outpatient Department of Surgery, Shandong Provincial Hospital Affiliated to Shandong First Medical University, No. 324, Jingwu Road, Huaiyin District, Jinan, Shandong 250021, China; Outpatient Department of Surgery, Shandong Provincial Hospital Affiliated to Shandong First Medical University, No. 324, Jingwu Road, Huaiyin District, Jinan, Shandong 250021, China; Outpatient Department of Surgery, Shandong Provincial Hospital Affiliated to Shandong First Medical University, No. 324, Jingwu Road, Huaiyin District, Jinan, Shandong 250021, China

**Keywords:** kidney stone, kidney transplantation, ultrasound-guided, percutaneous nephrolithotomy, percutaneous ureteroscopic lithotomy, new biological

## Abstract

A 39-year-old man was admitted to our hospital with kidney stones after kidney transplantation. Kidney, ureter, and bladder radiographs showed multiple stones in the transplanted and orthotopic kidneys, which had not been reported previously. Owing to the larger size of the stones in the transplanted kidney, they needed to be removed. Percutaneous nephrolithotomy and ureteroscopy were performed under B-mode ultrasound guidance. The stone measured 1.9 × 1.6 cm and was located under the calyx of the kidney. A titanium laser fiber was used to dissolve the stones, which were subsequently removed. No adverse reactions occurred during or after the surgery. The causes of stone formation included dietary factors, related drugs, improper fluid intake, and urinary tract infections. As neither the donor nor the recipient had a history of kidney stones, we hypothesized that the stones were a new entity that either developed following transplantation or a long-term complication.

## Introduction

With the increasing popularity of kidney transplantation, the number of people receiving kidney transplantation has been increasing, leading to an increase in the incidence of kidney stones. Removal of kidney stones is particularly important because of the potential risk of obstruction, sepsis, and even allograft kidney loss [1]. Although there are many methods to treat renal calculi after kidney transplantation, there is no uniform standard of treatment, and there are many uncertainties about the etiology of renal calculi following kidney transplantation [2, 3]. Herein, we present a safe and effective method for ultrasound-guided percutaneous nephrolithotomy and ureteroscopic lithotomy. We believed that the cause of stone formation was related to the patient’s stone constitution.

**Figure 1:**
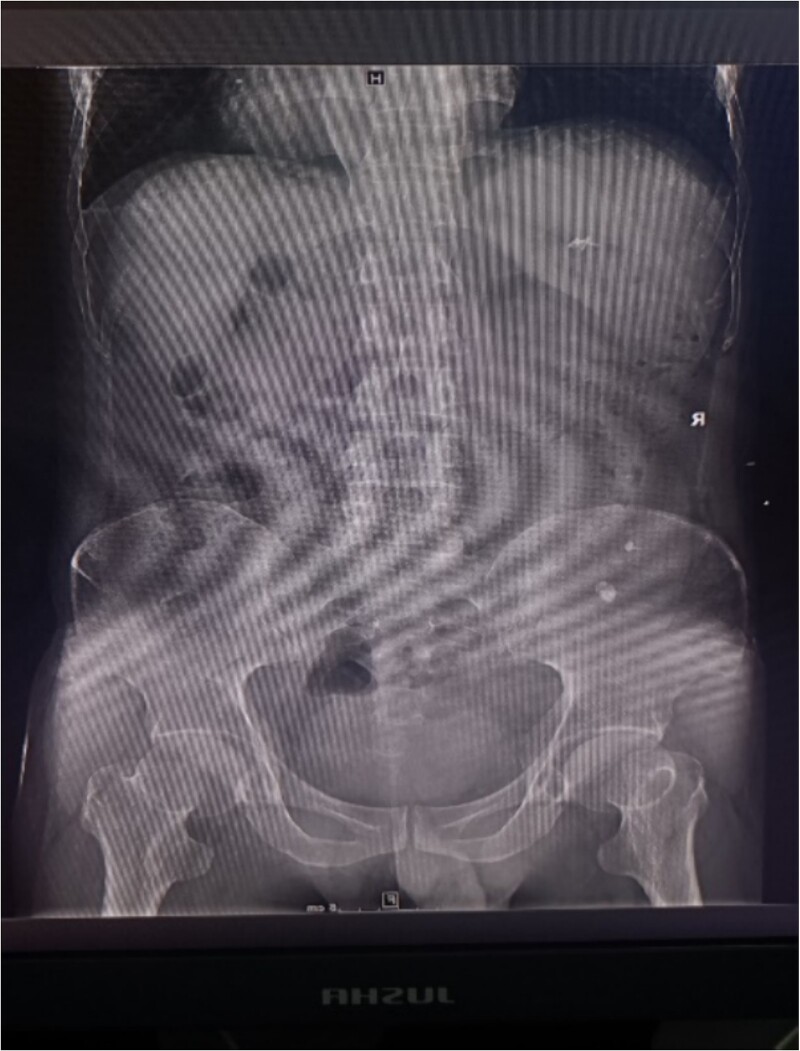
X-ray plain film showing multiple punctate stones in the right kidney area.

**Figure 2:**
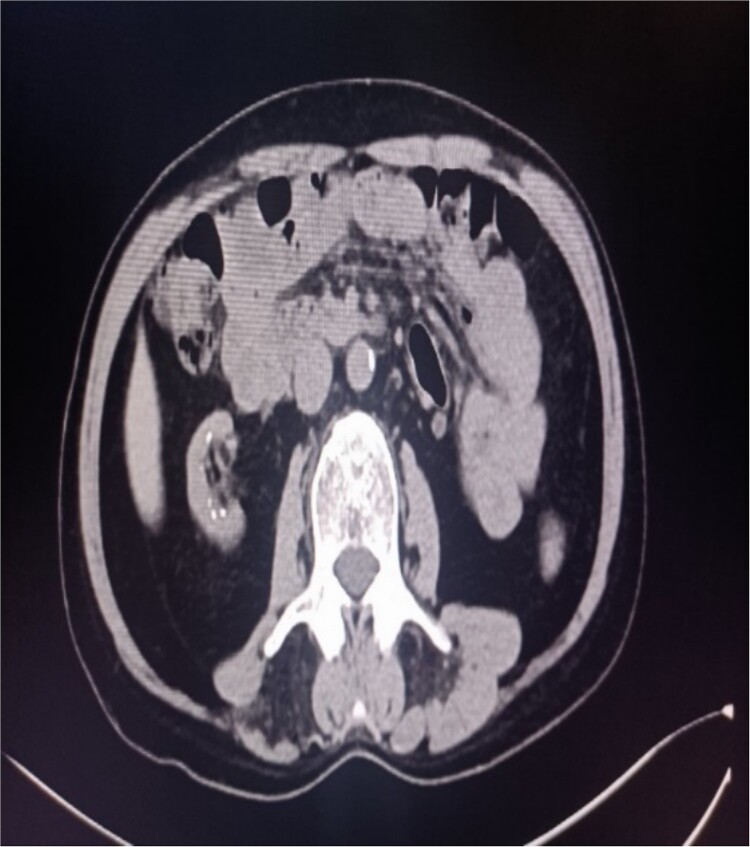
Abdominal CT showing multiple punctate stones in the right renal area *in situ*.

**Figure 3:**
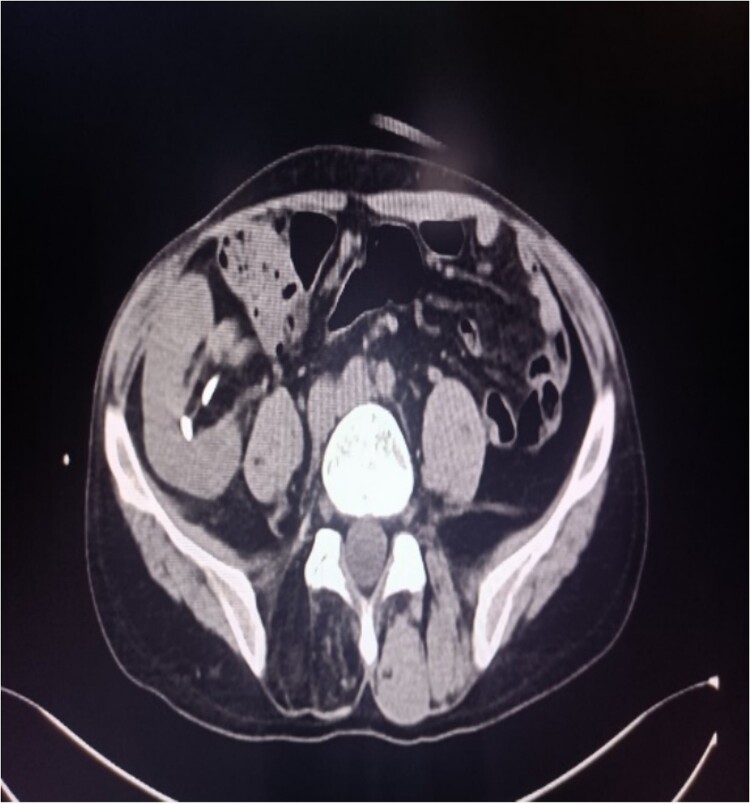
CT of the transplanted kidney shows stones.

## Case report

A 39-year-old man with kidney stones after kidney transplantation was admitted to our hospital on 29 November 2023. Eight years ago, he underwent a kidney transplant from a donor. Neither the donor nor the recipient had a history of kidney stones. The transplanted kidney was positioned in the right iliac fossa, and the patient had a successful postoperative recovery. However, a recent routine abdominal computed tomography (CT) scan revealed multiple stones in the transplanted kidney and bilateral *in situ* kidney atrophy. The stone was located in the lower calyx of the transplanted kidneys. Hydronephrosis was also observed. Kidney, ureter, and bladder radiographs showed multiple high-density speckles in the right kidney, the largest of which was ~0.5 cm in diameter. Physical examination (blood pressure, 147/91 mmHg; serum creatinine, 72.7 μmol/L; urea nitrogen, 5.1 mmol/L) revealed normal liver function, coagulation, and electrolytes. We suspected that these kidney stones were sizable and necessitated surgical intervention. Upon admission, pertinent examinations were conducted, and preoperative preparations were completed.

**Figure 4:**
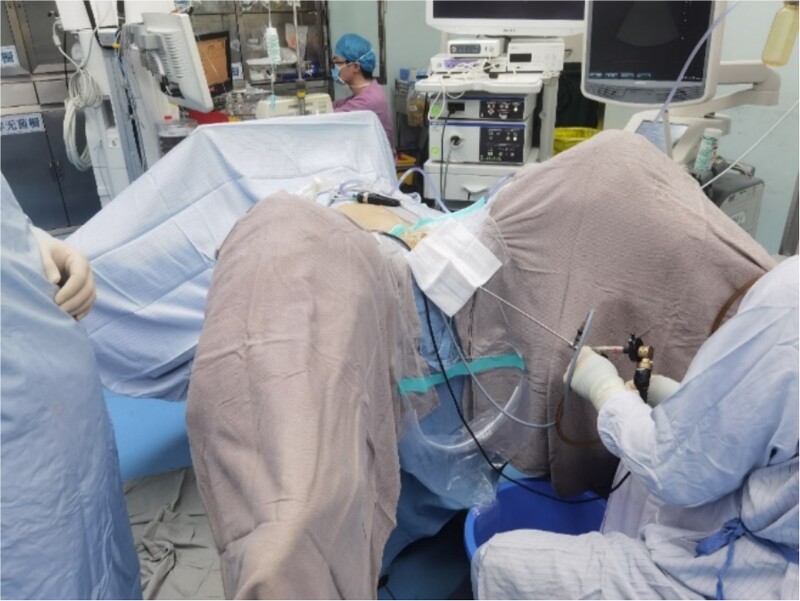
Percutaneous ureteroscopic lithotripsy.

**Figure 5:**
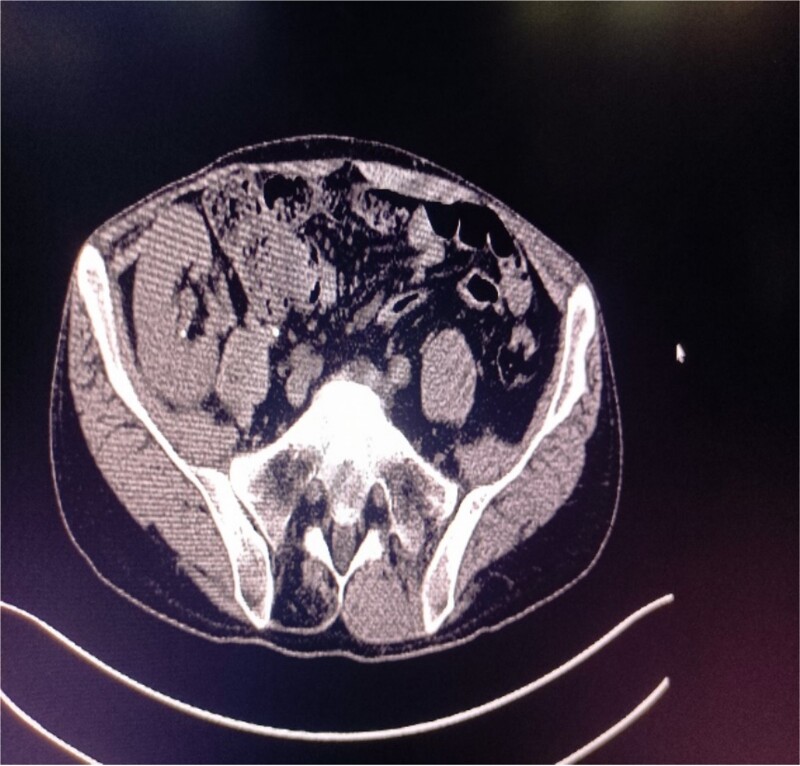
After removal of stones in the transplanted kidney.

After successful general anesthesia, the patient was placed in the lithotomy position. Initial ureteroscopy revealed no visible stones or tumors in the bladder. Both ureters were closed and replanted on the upper right wall. However, owing to the degree of opening, a zebra guidewire and stent could not be implanted. A percutaneous nephrolithotomy was performed. Under B-mode ultrasound guidance, the F20 tri-lumen catheter and hook guidewire were inserted into the upper calyx of the transplanted kidney. The polytetrafluoroethylene expanders along the guidewire were expanded and equipped with an F18 stripper, obscuring the visibility of the lower calyx under a rigid ureteroscope. The size of the lower calyceal stone, measured using the flexible ureteroscope, was 1.9 × 1.6 cm. The mucosa around the stone showed hyperemia and edema. After implanting a titanium laser fiber, the stone was crushed and a stone basket was used to corral the larger stones to the outside of the body. Subsequently, two F6.0 double-J tubes were implanted into the ureters, and the F14 nephrostomy tube was placed through the renal puncture channel to drain the ureters. The surgery was successful without any adverse reactions. No residual stones were detected in the transplanted kidneys, and renal function was improved.

Although the incidence of kidney stones after kidney transplantation is only 0.17%–4.40%, kidney stones can cause obstruction, sepsis, and even allograft kidney loss. Treatment of these stones continues to pose a significant challenge, as the objectives include eliminating stones, enhancing renal function, minimizing damage to the transplanted kidney, and prolonging its life span.

## Discussion

### Percutaneous ureteroscopic lithotripsy

Given recent advancements in urinary system diagnosis and treatment, diverse techniques such as microchannel percutaneous nephrolithotomy and ureteroscopic lithotripsy have become viable for treating kidney stones after kidney transplantation [2]. We evaluated the efficacy of various treatments, including extracorporeal lithotripsy, open surgery, percutaneous nephrolithotomy, and ureteroscopy. Open surgery, percutaneous nephrolithotomy, and ureteroscopy yielded the highest stone-free rates [2]. Each aggressive treatment approach is effective in terms of stone clearance, emphasizing the need to tailor surgical techniques according to the patient’s characteristics and the surgeon’s preference [4].

### Percutaneous nephrolithotomy

This is a risky and challenging kidney procedure. Under the guidance of B-mode ultrasound, the whole kidney can be scanned, a stereogram can be obtained, the stone position can be observed in real time, the relationship between the puncture needle and collection system can be monitored in real time, and the size of the transplanted kidney and blood flow in the artery and vein can be observed dynamically using a simple, noninvasive, and highly accurate operation [5]. Zhang and Gao [6] reported that for patients with multiple calculi in the lower calyx and ureter after kidney transplantation, using multiple combined approaches effectively addresses specific anatomical challenges when a single approach is not.

## Occurrence of complications

Renal allograft calculus formation is a rare complication, with a mean duration of 1.6–3.5 years and a prevalence of 0.2%–6.3% [7]. Approximately 2% of renal transplant recipients are diagnosed with kidney stones within 3 years of transplantation, and the incidence of urolithiasis ranges from 0.1% to 6.3%, usually diagnosed 12 months after kidney transplantation [8]. Kidney stones are typically diagnosed ~1 year after surgery, mainly in the calyces and renal pelvis, with the main component being calcium oxalate [4]. In this case, 8 years after renal transplantation, abdominal imaging revealed multiple stones in the transplanted kidney with renal stones *in situ*. However, this unique situation has not been reported previously. The most common type of kidney stone in transplanted kidneys is calcium-based, followed by guano and uric acid stones [9]. Calcium oxalate is the most common constituent, followed by uric acid [3]. Risk factors for kidney stone formation include a history of kidney stones, long-term dialysis, and conditions such as gout and hypertension [8]. Additionally, dietary habits, certain medications, inadequate fluid intake, urinary tract infections, hyperparathyroidism, and hypercalcemia are associated with an increased risk of kidney stones after kidney transplantation [9, 10]. In this particular case, neither the donor nor the recipient had a history of kidney stones. Thus, we infer that kidney stones following kidney transplantation represent a novel entity or a long-term complication associated with the patient’s predisposition to stone formation. This offers a fresh perspective on the development of renal allograft stones.
